# Pituitary Hormones and Orofacial Pain

**DOI:** 10.3389/fnint.2018.00042

**Published:** 2018-10-02

**Authors:** Gregory Dussor, Jacob T. Boyd, Armen N. Akopian

**Affiliations:** ^1^School of Behavioral and Brain Sciences, University of Texas at Dallas, Richardson, TX, United States; ^2^Department of Cellular and Integrative Physiology, University of Texas Health Science Center at San Antonio, San Antonio, TX, United States; ^3^Department of Endodontics, University of Texas Health Science Center at San Antonio, San Antonio, TX, United States; ^4^Department of Pharmcology, University of Texas Health Science Center at San Antonio, San Antonio, TX, United States

**Keywords:** pituitary, hormones, orofacial pain, headache, migraine, nociception

## Abstract

Clinical and basic research on regulation of pituitary hormones, extra-pituitary release of these hormones, distribution of their receptors and cell signaling pathways recruited upon receptor binding suggests that pituitary hormones can regulate mechanisms of nociceptive transmission in multiple orofacial pain conditions. Moreover, many pituitary hormones either regulate glands that produce gonadal hormones (GnH) or are regulated by GnH. This implies that pituitary hormones may be involved in sex-dependent mechanisms of orofacial pain and could help explain why certain orofacial pain conditions are more prevalent in women than men. Overall, regulation of nociception by pituitary hormones is a relatively new and emerging area of pain research. The aims of this review article are to: (1) present an overview of clinical conditions leading to orofacial pain that are associated with alterations of serum pituitary hormone levels; (2) discuss proposed mechanisms of how pituitary hormones could regulate nociceptive transmission; and (3) outline how pituitary hormones could regulate nociception in a sex-specific fashion. Pituitary hormones are routinely used for hormonal replacement therapy, while both receptor antagonists and agonists are used to manage certain pathological conditions related to hormonal imbalance. Administration of these hormones may also have a place in the treatment of pain, including orofacial pain. Hence, understanding the involvement of pituitary hormones in orofacial pain, especially sex-dependent aspects of such pain, is essential to both optimize current therapies as well as provide novel and sex-specific pharmacology for a diversity of associated conditions.

## Introduction

Orofacial pain conditions dramatically impact basic daily life activities such as talking and chewing, which leads to debilitating physiological and psychological consequences (Murray et al., 1996). Many conditions that localize to the orofacial region present with sex-specific differences in prevalence, chronicity and severity of pain (Mogil, 2012). Trigeminal-regulated orofacial pain conditions include temporomandibular disorders (TMDs), migraines, cluster headaches, tension-type headaches, burning mouth syndrome, neuralgias, dental-associated conditions, head and neck cancers and certain idiopathic pain syndromes. Women present with a 50% (TMD and tension-type headache), 90% (burning mouth syndrome) and up to 200% (migraine) increase in orofacial pain frequency over men (Cairns, 2007; Shinal and Fillingim, 2007). In contrast, men develop cluster headaches at nearly five times the rate of women, demonstrating the vast sexual dimorphism seen in orofacial pain (Holroyd et al., 2000; Shinal and Fillingim, 2007). The prevalence of chronic pain among women was also reported in a large epidemiological study, which compared orofacial with other types of pain (Fillingim et al., 2009). Overall, chronic orofacial pain occurs in about 10% of adults and 50% of the elderly, and of those with orofacial pain lasting more than a week, over 90% complain of pain in other areas of the body (Madland et al., 2001; Kohlmann, 2002; Mogil, 2012).

The higher prevalence of chronic pain in female patients could be explained by: (1) females could have different pain tolerance/thresholds than men and they may also seek out health care services at higher rates than do men. While there is evidence that this can be the case for humans, animal data do not convincingly show differences in tolerance/threshold between sexes so it may be unlikely that sensitivity alone explains higher female pain prevalence (Mogil, 2012); and (2) there are apparent sex differences in the responsiveness, tolerance, pharmacokinetics and/or pharmacodynamics for many analgesics, especially opioids (Niesters et al., 2010; Campesi et al., 2012; Franconi and Campesi, 2014).

A third factor, and the subject of this review article, is that the distinct sexual differences seen in orofacial pain conditions are mediated by hormone-based mechanisms. In fact, changes in gonadal hormones (GnH) such as estrogen, progesterone and androgens are shown to be associated with changes in pain experience in many orofacial pain conditions (Shinal and Fillingim, 2007). For instance, women using exogenous hormones report more severe orofacial pain compared to women not using hormones (Wise et al., 2000). Similarly, pregnant women during the first trimester experience a dramatic increase in pain associated with periodontal disease, gingivitis, caries and erosions (Kandan et al., 2011). Women also report that noxious chemical (capsaicin, serotonin or glutamate) injection into the facial skin or into the masseter muscle evokes more significant pain than in men (Cairns, 2007). Animal studies corroborate the hormone-dependency of this effect since estrogen replacement therapy in male or ovariectomized (OVX) female rats increases excitability of neurons innervating the TMJ and also increases the magnitude of glutamate-evoked jaw muscle nociception (Cairns et al., 2002; Flake et al., 2005). Moreover, expression and functions of many hormones, including prolactin (PRL), growth hormone (GH) and thyroid hormones, are influenced by analgesics (Mistraletti et al., 2005; Merza, 2010; Vuong et al., 2010; Gudin et al., 2015). The mechanisms that underlie the hormonal and sex-differences seen in prevalence, intensity and especially chronicity of orofacial pain and nociception are still not clear. Nevertheless, this area of pain research is experiencing rapid advances and the currently-available information and existing hypothesis will be reviewed here. The many studies on the influence of analgesics on the function of hormones are covered elsewhere (Demarest et al., 2015; Gudin et al., 2015).

While extensive research has looked at GnH regulation of pain, the impact of GnH-regulating hormones like gonadotropin releasing hormone (GnRH), PRL, follicle stimulating hormone (FSH), luteinizing hormone (LH) and other pituitary hormones has not been explored to the same depth. Hormones produced by the pituitary were originally named and characterized according to their primary biological function at the time of discovery: PRL is linked to milk production in females, GH is associated with cell growth, proliferation, differentiation and regeneration and other pituitary hormones, such as FSH, LH and adrenocorticotropic hormones (ACTHs) are master controllers of critical glands. Later studies established that pituitary hormones play critical roles in a much wider variety of physiologic and pathophysiologic processes. Thus, many of pituitary hormones have been associated with pain conditions across the entire body. This review article focuses on several pituitary hormones which according to human and animal studies are known to be involved in the regulation of orofacial pain. Particular emphasis will be placed on hormones regulating GnH production or those regulated by GnH, since they are viable candidates for the sexually-dimorphic regulation of orofacial pain.

## Prolactin

The main variant of PRL is a 23 kDa protein (Ben-Jonathan et al., 2008). Pituitary production of PRL is closely regulated by estrogen via an estrogen-response element found in its promoter. In addition, PRL elevation down-regulates the sex hormones (GnH) estrogen and testosterone (discussed below; Grattan et al., 2007). PRL production and release by the pituitary is modulated by many factors, including hormones, stress and trauma (Freeman et al., 2000). The main regulator of PRL secretion from pituitary (Pit PRL) is dopamine, which is released from tuberoinfundibulum (TIDA) neurons of the arcuate nucleus and acts on the D2 receptors of lactotrophs (pituitary cells producing PRL), inhibiting Pit PRL release (Freeman et al., 2000). PRL is also produced by several extra-pituitary tissues (EPit PRL) and can act through paracrine and autocrine mechanisms (Ben-Jonathan et al., 1996). PRL performs its biological function by activating the PRL receptor (Prlr), which is widely expressed in many cell types (Mancini et al., 2008). Prlr belong to the cytokine-class 1 receptor family, is encoded by one gene and has two main forms: long (Prlr-L) and short (Prlr-S; Freeman et al., 2000). Prlr-L predominantly signals through the JAK-STAT5 pathway, regulates transcription and produces long-lasting effects (Brown et al., 2012; Yip et al., 2012). In contrast, activation of Prlr-S produces transient effects through the PI3K/PKC pathway but is not capable of inducing the JAK-STAT5 pathway (Belugin et al., 2013). Prlr in humans (or primates) is distinct from rodent Prlr in one important aspect; it is activated not only by PRL, but also by GH and placental lactogen (Ben-Jonathan et al., 2008). This type of cross-reactivity of Prlr in humans is important for determining disease mechanisms and also developing potential therapeutics.

Pituitary adenomas are classified as nonfunctional (silent) or functional (hormone secreting) with symptomology dependent on the specific hormone(s) secreted. Headache and facial allodynia are common in patients with functional adenomas (Abe et al., 1998; Levy et al., 2005), especially PRL-secreting tumors (prolactinomas or hyperprolactinemia). Patients typically present with sexual dysfunction, galactorrhea and highly elevated PRL in serum (normal 1–20 ng/ml vs. prolactinomas 40–2,000 ng/ml (Kallestrup et al., 2014). Prolactinoma-induced headache has been classified as migraine-like (Hartman et al., 1995) with trigeminal autonomic cephalalgias, including cluster headache (Porta-Etessam et al., 2001; Negoro et al., 2005), paroxysmal hemicrania (Sarov et al., 2006) and short-lasting unilateral neuralgiform headache attacks with conjunctival injection and tearing (SUNCT; Matharu et al., 2003; Chitsantikul and Becker, 2013). Headache associated with prolactinomas can be effectively treated with dopamine agonists, which block PRL secretion from the pituitary (Hartman et al., 1995; Gabrielli et al., 2002; Kallestrup et al., 2014). Migraineurs without pituitary adenomas do not have higher serum PRL levels compared to controls (Guldiken et al., 2011); however, PRL rises during migraine attacks but not tension-type-headaches (Di Mario et al., 2006; Bosco et al., 2008). Moreover, some cases of migraine have been successfully treated by blocking pituitary PRL with the dopamine agonist—bromocriptine or carbidopa/levodopa (Gordon et al., 1995; Hartman et al., 1995).

The precise mechanism(s) for how elevated endogenous PRL causes pain is not fully known, but clear links between PRL and the somatosensory system exist. Prlr is known to be expressed in a subset of rat and mouse sensory neurons of dorsal root ganglia (DRG) and trigeminal ganglia (TG) neurons, although the precise Prlr^+^ sensory neuron sub-type identity is still unresolved (Table 1; Diogenes et al., 2006). Nevertheless, exogenous PRL transiently potentiates transient receptor potential V1 (TRPV1) in rat TG neurons (Diogenes et al., 2006), and TRPV1, TRPA1 and TRPM8 in mouse DRG neurons (Belugin et al., 2013; Patil et al., 2013b). TRPA1 and TRPM8 regulation by PRL in TG neurons were not yet tested. PRL sensitizes TRPV1 in mouse DRG neurons by activating Prlr-S via PKCδ and PI3-kinase pathways (Belugin et al., 2013; Patil et al., 2013b). Importantly, 40-fold lower concentration of PRL is required to substantially sensitize TRPV1 in female (>25 ng/ml) than male (>1 μg/ml) mouse and rat DRG and TG neurons (Diogenes et al., 2006; Belugin et al., 2013; Patil et al., 2013b). Behavioral experiments also show exogenous PRL can induce acute nociception in cycling female, but not OVX rats (Diogenes et al., 2006). This indicates that Prlr displays estrogen-dependent activity in rodent sensory neurons. However, whether human sensory neurons show sex-dependency in Prlr expression, and whether sensory neuronal Prlr contributes to pain in a variety of models (or diseases), and the precise mechanisms underlying female-selective activity of Prlr and GnH regulation of pain, remain unidentified.

**Table 1:** Expression of pituitary and gonadal hormones (GnH), their releasing hormones and receptors in male mouse dorsal root ganglia (DRG) sensory neurons.

Hormone system	Protein/peptide name	Gene name	Expression level (RPKM)	Expression pattern
Prolactin	Prolactin	PRL	1–2	C-fiber peptidergic and mrgA3^+^ neurons
	Prolactin receptor	Prlr	6–40	In C- and A-fiber peptidergic neurons and PV^+^ neurons. Prlr was nor revealed in mouse male TG neurons (Lopes et al., 2017)
Oxytocin	Oxytocin	OXT		Very low level (3.777 RPKM) in trkB^+^ neurons
	Oxytocin receptor	OXTR	2–3	trkB^+^ and PV^+^ neurons. OXTR is expressed in peptidergic DRG neurons (Tzabazis et al., 2016; Lopes et al., 2017)
Somatotropin	Growth hormone	Gh		None
	Growth hormone receptor	Ghr	13–54	Almost all sensory neurons
	Growth hormone-releasing hormone	Ghrh		None
	Growth hormone releasing hormone receptor	Ghrhr		None
	Growth hormone-inhibiting hormone	SST	1,395	TRPV1^+^/CGRP^−^ neurons (NP-3)
			9–20	mrgA3^+^ and C-fiber peptidergic neurons
	Growth hormone-inhibiting hormone receptor	Sstr1	2–5	mrgA3^+^, C-fiber peptidergic and trkB^+^ neurons
Corticotropin	Adrenocorticotropin (ACTH)	POMC	2–10	Almost all neurons
	Corticotropin releasing hormone	Crh		None
	Corticotropin releasing hormone receptor	Crhr1	3.5	PV^+^ neurons
	ACTH receptor—MC2R	Mc2r		None
	Glucocorticoid receptors	NR3C1	10–100	Almost all neurons
Thyroid	Thyroid-stimulating hormone (beta subunit)	Tshb		None
	Thyroid-stimulating hormone receptor	Tshr	15	A-fiber peptidergic neurons
	Thyrotropin-releasing hormone	Trh		None
	Thyroid-releasing hormone receptor	Trhr	6	Large neurons (NF groups)
		Trhr2		None
	thyroid hormone receptor	Thra		None
		Thrb	1–10	Almost all neurons
Gonadotrophins	Luteinizing hormone (beta)	Lhb		None
	Follicle stimulating hormone (beta subunit)	Fshb		None
	Gonadotropin-releasing hormone	Gnrh1		Almost none
	Gonadotropin releasing hormone receptor	Gnrhr	3–4	mrgA3^+^ and C-fiber peptidergic neurons
Gonadal hormones	Estrogen receptor α	Esr1	2–10	All neurons
	Estrogen receptor β	Esr2	10	A-fiber peptidergic neurons
	GPR30	Gpr30	20	TRPV1^+^/CGRP^−^ neurons (NP-3)
	Progesterone receptor	Pgr	1–10	C-fiber peptidergic and large neurons
	Testosterone receptor	Ar	3–10	C and A-fiber nociceptors
			10–60	Large neurons (NF groups)

*Data is from Usoskin et al. (2015)*. *PV is parvalbumin*. *RPKM is Reads Per Kilobase Million. 1–3 RPKM—very low expression level; 3–10 RPKM—low expression level; 10–100 RPKM—moderate expression level; 100–1,000 RPKM—high expression level; >1,000 RPKM—very high expression level*.

As mentioned previously, sources of endogenous PRL can originate from pituitary (i.e., Pit PRL) or extra-pituitary (EPit PRL) tissues (Ben-Jonathan et al., 1996). It is well known that acute restraint stress induces Pit PRL in both female and male rodents (Brown et al., 2016). Pit PRL release is also increased by trauma and surgery in both humans and rodents (Noel et al., 1972; Anand, 1986; Chernow et al., 1987; Yardeni et al., 2007; Patil et al., 2013a). Stress and trauma-induced Pit PRL is slightly higher in females than males (Reiner et al., 1987; Patil et al., 2013a). In contrast, EPit PRL expression/release in relation to various pain conditions not as well studied and there are essentially no publications on humans or primates. Recent data in mice indicate that surgery and inflammation elevate local (i.e., hind paws) PRL and spinal cord PRL levels (Scotland et al., 2011; Patil et al., 2013a). Unlike Pit PRL, the increase in EPit PRL is profoundly female-specific (Scotland et al., 2011; Patil et al., 2013a). Sources of EPit PRL during pain conditions have not been fully explored, but multiple groups have speculated that it could be supplied by immune cells (Clevenger et al., 1990; Hawkins et al., 1996).

**Figure 1:**
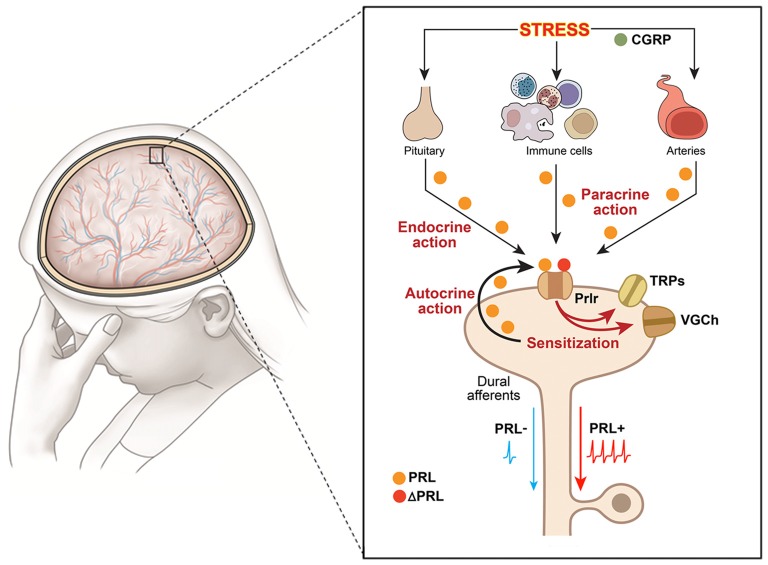
Schematic illustration of putative mechanisms underlying hyperalgesic action of the endogenous PRL system in orofacial pain conditions. Schematic shows an orofacial pain condition, i.e., migraine, triggered by stress. The presented pathway could be suggested for other orofacial conditions triggered by inflammation or trauma. The large figure represents dura mater with nerves and vessels running throughout, and the inset shows multiple pathways for PRL release and action on sensory neurons. Dural afferents are peripheral terminals of a subset of trigeminal ganglion neurons; VGCh, voltage-gated channels; TRP, transient receptor potential; Immune cells—PRL-expressing macrophages, mast and T cells as main candidates; Prlr, prolactin receptor; ΔPRL, Prlr antagonist, which is modified PRL that binds but does not activate Prlr; CGRP, calcitonin gene related peptide; PRL−, dural afferents without PRL stimulation; PRL+, dural afferents stimulated with PRL.

Endogenous PRL can contribute to female-selective mechanisms of postoperative and inflammatory nociception as shown in PRL and Prlr global knock-out mice (Patil et al., 2013a,b). Although the precise mechanisms underlying sex-dependent involvement of the PRL system in a variety of pain conditions is still unknown, a multi-step process can be inferred from the current literature. First, endogenous Pit and/or EPit PRL are elevated and released during stress, migraine attacks, trauma and inflammation-induced pain conditions (Figure 1). Second, endogenous PRL acts via endocrine, autocrine and/or paracrine pathways on sensory neurons through Prlr, transiently sensitizing TRP channels and possibly increasing excitability and/or neurotransmission to spinal cord (Patil et al., 2014; Figure 1). Finally, these changes generate thermal and mechanical hyperalgesia (Figure 1). The female-selectivity of the suggested pain mechanisms comes both from elevated PRL during stress or injury, and especially through the sexually-dimorphic and cell type-specific functional expression of Prlr in sensory neurons. Mechanisms underlying sex and GnH-dependent regulation of functional expression of Prlr in sensory ganglia are still unknown. But, a recent report indicates that Prlr mRNA expression can be detected in mouse male DRG, but not TG neurons (Lopes et al., 2017). Prlr mRNA was identified in both female DRG and TG neurons (Diogenes et al., 2006; Patil et al., 2013b). This may indicate that sex-dependent Prlr mRNA expression is more pronounced in TG than DRG neurons. As a consequence, PRL may preferentially contribute to sexual-differences in orofacial pain. However, sex-dependent contributions of the endogenous PRL in postoperative and inflammatory pain of hind paws were reported (Patil et al., 2013a,b). Accordingly, the PRL system could involve different sex-dependent mechanisms for spinal vs. orofacial pain. For instance, PRL may contribute to nociceptive transmission via indirect mechanisms. Endogenous PRL could activate Prlr on non-neuronal cells, leading to sensitization of sensory neurons via non-neuronal to neuronal cell interactions. Overall, the mechanisms critical to Prlr functional expressions in females vs. males and in TG vs. DRG need further exploration.

It is also unknown whether the PRL and Prlr sexual-dimorphism seen in mouse studies is applicable in primate and human sensory neurons. Prolactinomas provide a confounding example since severe headaches are seen equally in males and females with the tumors (Kallestrup et al., 2014). Extremely high levels of PRL can also cause sensory neuron sensitization in male mice, so the phenomenon seen in men with prolactinomas may be a result of the overwhelmingly exaggerated levels of PRL (Patil et al., 2013b). Another complication to high PRL resulting in increased pain is the issue of why pregnant in third trimester and lactating women do not report amplified pain experiences. One possible explanation is that pregnancy at the third trimester increases T cell-mediated endogenous opioid analgesia in the spinal cord (Rosen et al., 2017). This could counteract endogenous PRL’s hyperalgesia effects. There is strong interaction of the PRL and endogenous opioid systems in hypothalamic TIDA neurons (Tolis et al., 1978). Thus, endogenous opioid analgesia during pregnancy might still involve PRL, since endogenous opioids, acting via mu and kappa opioid receptors, suppress dopamine secretion from TIDA neurons, raising PRL levels in serum (Tolis et al., 1978). Prolonged elevation of PRL in serum of the third trimester pregnant women could suppress inflammation and pain (see below; Adán et al., 2013; Ledesma-Colunga et al., 2017). Moreover, systemic treatment with naloxone (a mu-opioid receptor antagonist) during late pregnancy completely blocks PRL secretion (Ieiri et al., 1980; Rossier et al., 1980). So the naloxone treatment used by Rosen et al. (2017) may affect not only the opioid but also the PRL system. Another potential explanation for the conflicting PRL effects during lactation could be the elevation of oxytocin (OXT) stimulated by breastfeeding (discussed below; Ren et al., 1997). Finally, prolonged and repetitive treatment with high concentrations of PRL (equivalent to the concentrations encountered during the third trimester of pregnancy) can suppress inflammation and pain (Adán et al., 2013; Ledesma-Colunga et al., 2017). Overall, further research on the function of the PRL system on the nociceptive pathway is vital for both determining mechanisms of sexual-dimorphism in orofacial pain and possibly developing new and sex-targeted therapeutics.

## Oxytocin

OXT is a peptide hormone (1 kDa), which is mainly produced by the paraventricular nucleus (PVN) of the hypothalamus and the posterior pituitary (D’Antoni, 2016). The precursor peptide contains OXT and the carrier protein neurophysin I (Brownstein et al., 1980). Elevation of OXT release above background levels depends on many factors and is regulated by estrogen (Acevedo-Rodriguez et al., 2015). There is a vast literature and multiple reviews on factors controlling OXT release, biosynthesis and degradation. Classical factors responsible for OXT release in the blood are: stretching of the cervix and uterus during labor and stimulation of the nipples from breastfeeding (Takeda et al., 1985). OXT fulfils its biological functions through activation of its receptor (OXTr; Gimpl and Fahrenholz, 2001). The OXTr, a 7-transmembrane G protein-coupled receptor capable of binding to either Gi or Gq proteins, can activate a set of signaling cascades, including the MAPK, PKC, PLC, or calmadulinK (CaMK) pathways, which converge on transcription factors like CREB or MEF-2 (Jurek and Neumann, 2018).

Clinical studies on abdominal hysterectomy for non-cancer indications compared to cesarean delivery show that childbirth is not associated with a high incidence of post-surgery chronic pain in humans (Brandsborg et al., 2007; Brandsborg, 2012; Khelemsky and Noto, 2012). Peripheral nerve injury shows similar hypersensitivity in non-pregnant and mid-pregnancy rats, but after delivery this hypersensitivity is partially reversed (Gutierrez et al., 2013b). Importantly, partial reversal of peripheral nerve injury pain does not happen in lactating rodent females in the absence of pups (Gutierrez et al., 2013b). Since labor and breastfeeding promote elevation of OXT in blood as well as cerebrospinal fluid (Gutierrez et al., 2013b) and since PVN afferents project to the spinal cord (Reiter et al., 1994; Eliava et al., 2016), it is hypothesized that exogenous OXT could be used as an anti-hyperalgesia drug in a variety of pain conditions (Breton et al., 2008; Gutierrez et al., 2013a,b; Boll et al., 2017). Indeed, direct administration of OXT into the spinal cord produced analgesia in a patient with intractable cancer pain (Madrazo et al., 1987). In chronic and high-frequency episodic migraineurs, 1 month of intranasal OXT administration reduced pain and significantly decreased the frequency of headaches (Tzabazis et al., 2017). In animals, OXT gene ablation leads to reduction of stress-induced analgesia (Robinson et al., 2002), while stimulated OXT release from rat PVN reduces hyperalgesia generated by loose ligature of the sciatic nerve (Miranda-Cardenas et al., 2006). direct administration of OXT into rat TG attenuated mechanical hypersensitivity due to partial ligation of the infraorbital nerve (Kubo et al., 2017), and intranasal OXT also attenuated thermal hypersensitivity of inflamed facial skin, TMJ nociception and mechanical allodynia in trigeminal neuralgia, facial incision and nitroglycerin-induced headache behavior, respectively (Tzabazis et al., 2017).

**Figure 2:**
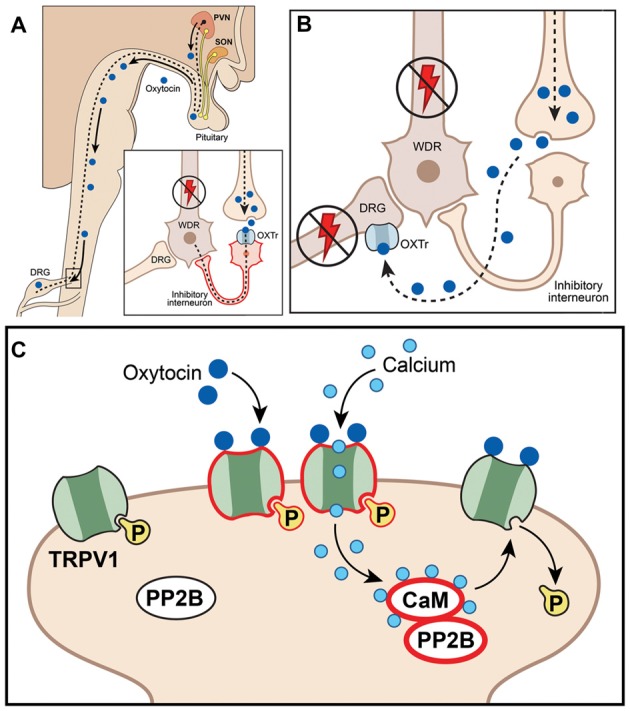
Schematic illustration of putative mechanisms explaining anti-hyperalgesic actions of the endogenous oxytocin (OXT) system in orofacial pain conditions. **(A)** Paraventricular nucleus (PVN) afferents projected to spinal cord release OXT, act on spinal neurons (including interneurons) and inhibit nociception; WDR, wide dynamic range spinal neurons; OXTr, OXT receptor; DRG, dorsal root ganglion. **(B)** OXT inhibits neurotransmission at DRG (or trigeminal ganglia, TG) by directly activating OXTr on central terminals, inhibiting activation and firing of dorsal horn wide-dynamic-range neurons. **(C)** OXT inhibits DRG (or TG) neurons by directly activating TRPV1, inducing Ca^2+^ influx and triggering desensitization mechanisms involving the calcineurin (PP2B)-calmadulin (CaM) pathway, which dephosphorylates TRPV1 and other channels (including VGChs).

While much is known underlying the anti-hyperalgesia effects of OXT, there is still debate as to the mechanism or mechanisms responsible. It is unknown whether OXTr signals through classical Gq or Gi pathways in sensory neurons, suggesting potentiating or inhibitory effects on sensory neuron functions (Boll et al., 2017). In addition, single-cell sequencing data indicate that OXTr is expressed at low levels in DRG neurons (Table 1; Usoskin et al., 2015). Three models for the anti-hyperalgesic actions of OXT have been proposed: the original descending model (Figure 2A), a peripheral OXTr model (Figure 2B), and a TRPV1 desensitization model (Figure 2C). The first model proposes a descending mechanism in which PVN afferents projecting to spinal cord release OXT upon electrical stimulation of PVN, labor or breastfeeding (Figure 2A). The released OXT activates a subset of lamina II glutamatergic interneurons (Figure 2A). These activated glutamatergic interneurons excite all GABAergic interneurons in lamina II, which in turn inhibits DRG-spinal cord neurotransmission of central terminals of incoming Aδ and C afferent sensory nerves (Figure 2A; Breton et al., 2008; Eliava et al., 2016). In later works, it was shown that GABAergic interneurons in lamina II are activated by OXT via an allopregnanolone pathway (Juif et al., 2013). The second model suggests direct inhibition of TG and DRG neurons by OXT through the OXTr (Figure 2B; Moreno-López et al., 2013; Tzabazis et al., 2017). According to this model, OXTr is expressed in the Aδ and C fiber central terminals of TG and DRG neurons (Moreno-López et al., 2013; Tzabazis et al., 2016). Spinal OXT is able to directly inhibit nociceptive neuronal firing in dorsal horn wide-dynamic-range neurons (González-Hernández et al., 2017). Using immunohistochemistry, it was shown that OXTr is expressed in male rat peptidergic TG neurons (Tzabazis et al., 2016) and OXT inhibits capsaicin-induced current in TG neurons and capsaicin-evoked calcitonin gene related peptide (CGRP) release from dura tissue (Tzabazis et al., 2016). Moreover, inflammation and electrical stimulation leads to rapid up-regulation of OXTr on TG neurons (Tzabazis et al., 2016). Precise mechanisms of how OXTr activation results in inhibition of sensory neurons, especially TRPV1 channels, are still unknown. It may be presumed that OXTr is a Gi-type receptor in TG and DRG neurons. This could explain the inhibition of neurotransmission (Boll et al., 2017), but it still does not explain the inhibition of TRPV1. Thus, activation of μ-opioid receptor signaling via the Gi pathway does not inhibit TRPV1-mediated current (Rowan et al., 2014). Another interesting observation from transcriptomic analysis is that OXTr mRNA is predominantly expressed on male TG compare to DRG neurons (Lopes et al., 2017). Since only low levels of OXTr are expressed in DRG neurons, a third model was developed to overcome discrepancies. It was suggested that OXT acts as a direct agonist of TRPV1 and produces anti-hyperalgesia effects via TRPV1 channel desensitization involving a calcineurin (a serine/threonine protein phosphatase) pathway (Nersesyan et al., 2017; Figure 2C). Alternative methods of OXT action on the nociceptive system have been proposed that involve either indirect modulation of TG and DRG neurons by OXT or the influence of OXT on emotional pain processing within the central amygdala (Eisenach et al., 2015). It is possible that a combination of these pathways accounts for the anti-hyperalgesic effects of OXT and may contribute to male vs. female pain states.

OXT shows sex-dependent effects in response to socially relevant stimuli (Theodoridou et al., 2013) and an increase in amygdala activity following threatening scenes (Lischke et al., 2012). However, OXT-induced inhibition of nociceptive pathways and analgesia were mainly studied in male rodents, demonstrating effective anti-hyperalgesia, suggesting less distinct sexual-dimorphic mechanisms than seen with PRL (Eliava et al., 2016; Tzabazis et al., 2016). One of the main complications in clinical studies with OXT is that it has a very short half-life (3–5 min) and does not readily cross the blood-brain barrier. This excludes oral or parenteral administration of exogenous OXT, leaving two viable possibilities: intra-spinal or intra-nasal (e.g., Syntocinon Nasal). Both approaches for OXT delivery produce short-living analgesia (Tzabazis et al., 2017). Therefore, analgesic efficacy of intranasal OXT in episodic migraineurs is relatively low and sometimes does not meet the primary endpoint of pain relief at 2 h (Tzabazis et al., 2017). Inflammation was found to play a critical role in upregulation of OXTr on rat TG neurons (Tzabazis et al., 2016), so a repeat study was performed in patients who had not taken anti-inflammatory drugs within 24 h. The results of this single-dose intranasal OXT application clinical study on chronic migraine patients was more encouraging and a follow-up 1 month intranasal study also showed a reduction in pain (Tzabazis et al., 2017). The results indicate that intranasal OXT might provide an effective therapy for migraine, although more studies still need to be performed.

## Growth Hormone

GH (aka somatotropin) is a 22 kDa protein produced by somatotrophs located in the anterior pituitary. GH production and secretion is regulated by myriad factors including hormones, sex hormones, stress, deep sleep, fasting, vigorous exercise, etc (Greenwood and Landon, 1966; Kanaley et al., 1997; Wren et al., 2000; Van Cauter et al., 2004). The primary mechanism for secretion of GH from the pituitary is regulated by GH-releasing hormone (GHRH; aka somatocrinin) and GH-inhibiting hormone (GHIH; aka somatostatin) produced by the neurosecretory nuclei of the hypothalamus (Lin-Su and Wajnrajch, 2002). GH is released in a circadian and pulsatory manner with the largest discharge happening after the onset of sleep and 50% of total daily release occurring during non-rapid eye movement sleep (Takahashi et al., 1968). Even though many of GH’s functions are still unknown, it is readily prescribed to treat children’s growth disorders (GHD) and adult GH shortage (Bright et al., 1999). GH signals through the GH receptor (GHr), which belongs to the cytokine receptor family, activates the JAK-STAT5 pathway, and is very closely related to Prlr (Brooks and Waters, 2010; Dehkhoda et al., 2018). Like Prlr, GHr can also signal transiently via the Src-PLCγ-PKC pathway (Brooks and Waters, 2010; Belugin et al., 2013).

Some pituitary adenomas dramatically increase secretion of GH (Kreitschmann-Andermahr et al., 2013). These patients reported a variety of types of headaches (Levy et al., 2005). Headaches improved dramatically in individual cases following successful surgery on removal of the tumor only to return with recurrent GH excess (Marzocchi et al., 2005). Additionally, somatostatin analogs rapidly eased headaches (Williams et al., 1987). In a large cohort of more than 30,000 children treated with GH for GHD, headache was reported with an incidence of 1%–3% patient years on initiation of GH therapy (Darendeliler et al., 2007). Circadian production of GH is shown to be disrupted in cluster headache patients, producing a bimodal profile with an abnormal evening peak during cluster periods (Leone and Bussone, 1993). Alterations in circadian GH secretion patterns were also reported in other headache syndromes (Ferrari et al., 1983). Excess GH produced by a functional pituitary adenoma could lead to a disorder called acromegaly. Two key symptoms of acromegaly are severe headache (Levy et al., 2005) and muscle pain (Khaleeli et al., 1984). Dural stretch and cavernous sinus invasion have been considered as mechanisms for acromegaly-induced headache. However, further studies showed that this may not be the case, since acromegaly-induced headache often occur without dural stretch and cavernous sinus invasion (Abe et al., 1998). Acromegaly can manifest the entire range of headache disorders: chronic and episodic migraine, SUNCT, cluster headache, primary stabbing headache, et cetera (Levy et al., 2008). The diversity of headache disorders occurring in acromegaly patients goes beyond any currently proposed mechanistic explanation. However, systemic treatment with a somatostatin analog results in dramatic improvement of pain-related acromegaly symptoms (Musolino et al., 1990). This suggests that decrease of GH results in reduction of headache in adult patients. Alternatively, it has been speculated that somatostatin inhibits the release of some, as yet undefined, pro-nociceptive peptide causing headache in patients with acromegaly (Williams et al., 1987). There are few additional reports that GH deficiency may indirectly lead to an increase in pain, specifically fibromyalgia-associated bone and muscle complications. It was shown that prolonged systemically applied GH therapy improves overall symptomatology, including the number of tender points in fibromyalgia patients (Bennett et al., 1998). It was also suggested that bone and muscle deficiencies may result in resting pain through GH deficiency (Bennett, 2004). Overall, a majority of clinical studies indicate that GH increase directly leads to severe pain, while few reports imply that GH shortage resulted in abnormalities indirectly affecting resting pain.

Regulation of pain pathways by the GH system is not well investigated, and there are only a few publications in this field. Some information indicates that GHr mRNA is expressed in neuronal cells, including spinal cord and DRG neurons (Table 1; Lobie et al., 1993; Kastrup et al., 2005). The specific subset of DRG neurons expressing functional GHr is still unknown, and GHr has also not been evaluated in TG neurons. So, it is not known whether GH could be able to transiently potentiate a subset of sensory neurons. Hind paw inflammation has been shown to reduce GH levels in affected skin of mice. Behavioral studies showed that like PRL (Adán et al., 2013), prolonged systemic treatment with high dose GH in inflamed (hind paw) mice leads to a reduction of mechanical and thermal hypersensitivity as well as excitability of C-fiber afferents (Liu et al., 2017). It has been suggested that this effect is due to GH’s action on inflammation-induced up-regulation of the insulin-like growth factor 1 receptor in mouse DRG neurons (Liu et al., 2017). Prolonged treatment of the rodent hindpaw with PRL does not change acute nociception and mechanical hyperalgesia. The effects on hyperalgesia and nociception after prolonged treatment in hind paw of naïve mice with GH are unknown. Alternative mechanisms involving epigenetic changes induced by GH have also not been considered and investigated as yet. Altogether, major research needs to be undertaken to further elucidate the underlying mechanisms governing modulation of the nociceptive pathway by the GH system.

## Adrenocorticotropic Hormone

Thirty nine amino acid-long (4.5 kDa) ACTH is a key component of the hypothalamic-pituitary-adrenal (HPA) axis, which is a chief controller of physiologic responses to both physical and emotional stress (Collip et al., 1933). ACTH coordinates neuroendocrine and autonomic responses through production and release of cortisol (i.e., glucocorticoid) by the adrenal glands. ACTH is a cleavage product of proopiomelanocortin (POMC), and is controlled by corticotrophin-releasing hormone (CRH), which is produced by the PVN of the hypothalamus in response to stress. The ACTH receptor (MC2R gene) is a Gs protein-coupled receptor that rapidly activates PKA upon stimulation (Hanukoglu et al., 1990). ACTH acting via MC2R also has long term actions by altering transcription of genes in a Ca^2+^-dependent manner (Raikhinstein and Hanukoglu, 1993). Single cell sequencing of lumbar DRG show MC2R is not expressed in sensory neurons (Table 1; Usoskin et al., 2015). However, ACTH can still regulate sensory neuronal plasticity through glucocorticoid production and downstream activation of glucocorticoid receptors (GR or NR3C1; Hollenberg et al., 1985). NR3C1 belongs to a nuclear receptor family controlling transcription (Hollenberg et al., 1985), it is expressed in almost all sensory neuronal groups, and it could directly influence sensory neuronal plasticity (Table 1; Usoskin et al., 2015).

Several well characterized pathological conditions are associated with misbalanced ACTH production or activity. Addison’s disease is a primary adrenal insufficiency most commonly arising from autoimmune disruption of adrenal response to ACTH (Brandão Neto and de Carvalho, 2014). Primary and secondary adrenal insufficiency result in severely decreased glucocorticoids and mineralocorticoids, but ACTH levels are elevated in primary and decreased in secondary diseases. There are many pain symptoms associated with both primary and secondary adrenal insufficiency, including myalgia, joint pain, sciatic-like pain and low back pain (Sheridan et al., 1976; Calabrese and White, 1979; Zaleske et al., 1984; Mor et al., 1987; Tzoufi et al., 2013). Because both primary and secondary adrenal insufficiencies present with similar pain phenotypes and starkly different ACTH levels, the symptoms appear to be due to glucocorticoid and/or mineralocorticoid deficiency (discussed below) and not the direct result of ACTH. Conversely, Cushing’s syndrome is characterized by overproduction of cortisol. Cushing’s can be triggered by exogenous corticosteroid use (↓ ACTH), primary adrenal tumors (↓ ACTH) or secondary pituitary tumors (↑ ACTH; Nieman and Ilias, 2005). Cushing’s patients were initially reported to display painful adiposity, but later characterizations revealed the syndrome seldom affects resting pain or nociception (Plotz et al., 1952). Again, the diversity of ACTH levels with no change in nociception indicates little direct role of ACTH on pain.

It is well known that stress is a trigger for many pain conditions, including migraine, TMJD and neuropathies. Cluster headache patients have also been found to have significantly elevated 24 h cortisol production in the attack (i.e., cluster) periods (Leone and Bussone, 1993). It was originally suggested that stress, due to pain, elevates cortisol levels. Animal studies showed acute stressor-induced activation of the HPA axis transiently suppressed pain and the inflammatory response (Brandt et al., 1976; Harmsen and Turney, 1985; Rhen and Cidlowski, 2005). Other evidence indicates that repeated stress (even mild) worsens nociception in a number of chronic inflammatory conditions and could also trigger development of nociception in naïve animals (Zautra et al., 1994). It was shown that repeated restraint stress on male rats aggravates inflammation via the adrenal cortex but not through adrenal medulla innervation-mediated mechanisms (Strausbaugh et al., 1999). Interestingly, this effect was mimicked by repeated systemic injections of corticosterone (Strausbaugh et al., 1999). Mechanisms responsible for this repeated corticosterone-triggered enhancement of inflammation are not clear and are controversial, since elevated cortisol at Cushing’s syndrome patients does not produce a pain phenotype. Basically, the unanswered question is whether cortisol directly regulates nociceptive pathways, whether cortisol induces nociception as a consequence of inflammation, which leads to up-regulation of inflammatory mediators sensitizing the nociceptive pathways, or whether other stress-induced proteins contribute to nociception more than cortisol (Green et al., 1995).

Stress may also regulate inflammation through sympatho-adrenal modulation of the inflammatory response. Miao et al. (1992) and Lundeberg et al. (1993) determined α2 adrenergic receptor activation decreases inflammation whereas β2 adrenergic activity increases chronic inflammation in male rats (Miao et al., 1992; Lundeberg et al., 1993). Many pain disorders associated with the sympathetic nervous system also show a female predominance, including complex regional pain syndrome (CRPS), formerly known as sympathetically maintained pain and causalgia (Berkley, 1997). Alternatively, stress-induced hyperalgesia may result from changes in the serotoninergic, nicotinic or opioid systems, which could explain the link between stress and orofacial pain (Miao et al., 1996; Gameiro et al., 2006). One group suggests that diurnal exacerbations of mechanical allodynia in neuropathy may be the result of glucocorticoid-induced release of ATP from spinal astrocytes, acting on microglia (Koyanagi et al., 2016). The diurnal rhythm of glucocorticoid release and allodynia may be dependent on ACTH release patterns independent of inflammation. However, the HPA axis does not show clear effects on pain conditions in which stress and inflammation are not present. For instance, hypophysectomy does not alter postoperative pain responses in females or males (Green et al., 2016). Surgery briefly upregulates ACTH (Srinivasan et al., 2011), but this does not enhance surgery-triggered inflammation. Likewise, it was found that long-lasting injury generated by nerve damage alters the limbic system but is dissociated from HPA axis activation (Ulrich-Lai et al., 2006). In summary, the sympatho-adrenal axis could play a more dominant role in stress-induced hyperalgesia than the pituitary. Nevertheless, this model presumes that stress-induced pain is controlled by stress hormones (i.e., cortisol, ACTH etc.), but stress can dramatically upregulate other pituitary hormones such as GH and PRL (see above).

Are acute and repeated stress-induced alterations in nociception and pain sex-dependent? After decades of research, there is an agreement that clear sex differences in the HPA stress system and its responses exist (Chen et al., 2014; Goel et al., 2014). The sympatho-adrenal stress axis, which is indirectly controlled by ACTH, is also sexually dimorphic (DeTurck and Vogel, 1980; Livezey et al., 1985; Green et al., 1999). HPA and sympatho-adrenal axes mediate key differences in GnH levels (Kudielka and Kirschbaum, 2005). For instance, activation of the HPA axis leads to suppression of the LH/testosterone/E2 pathways (Tsigos and Chrousos, 2002). Nevertheless, there is no consensus on whether stress exacerbates nociceptive and pain conditions more in animal and human females or males (Kudielka and Kirschbaum, 2005; Goel et al., 2014). The majority of studies imply that the HPA axis in females responds more rapidly to stress and produces a greater output of ACTH (Videlock et al., 2016). Long et al. (2016) showed that restraint stress affects formalin-induced mechanical hypersensitivity in male, but not female mice (Long et al., 2016). One reason for variation in results is the complex interaction between sites controlling the HPA axis, including POMC neurons of the arcuate nucleus, PVA and the central nucleus of the amygdala (CeA), which releases CRH depending on emotional state (Kastrup et al., 2005). Another reason for variable results is that the HPA axis is affected by other medications used in clinical settings, i.e., opiates and NSAIDs (Aloisi et al., 2011). Overall, the sexual-dimorphic role of the HPA axis and modulatory sites, such as POMC neurons and CeA, in pain conditions needs further study to establish a unified theory.

## Thyroid-Stimulating Hormone

Thyroid-stimulating hormone (TSH) controls tissue metabolism via production of thyroxine (T4) through iodination of thyroglobulin in thyroid gland follicles. T4 is later converted into the active hormone triiodothyronine (T3) at target tissues, and acts via a combination of transport and nuclear receptors (Brent, 2012). Release of TSH from the pituitary is positively regulated by hypothalamic thyrotropin-releasing hormone (TRH), while it is suppressed by somatostatin. TSH is also controlled by negative feedback of T3 and T4 at the anterior pituitary. TSH has two subunits, the alpha (92 AA) and the beta (118 AA). The TSH receptor (TSHr) is a G protein-coupled receptor that can act through both Gs and Gq mechanisms (Farid and Szkudlinski, 2004).

Several diseases are characterized by misbalanced TSH, T3 and/or T4. All forms of thyroid disease are at least 3–4 times more prevalent in women than in men (Gessl et al., 2012). Graves’ disease is the most common cause of hyperthyroidism. Graves’ presents with elevated T3 and T4, but decreased TSH due to autoimmune TSHr-stimulating IgG (Burch and Cooper, 2015). Graves’ disease presents with many ophthalmic and dermatologic symptoms, but pain thresholds are not affected in patients with this condition. Hashimoto’s is an autoimmune hypothyroid disease characterized by low T3 and T4, and high TSH. Thyroid hormone resistance is another hypothyroid disease that results from mutations in thyroid receptors. It is diagnosed with high T3, T4 and TSH but hypothyroid symptoms result from lack of receptor recognition. Unlike hyperthyroidism, hypothyroid patients with thyroid gland hormone (i.e., T3 and T4) deficiencies have significantly higher nociceptive thresholds (i.e., lesser pain) than control subjects (Guieu et al., 1993; Guasti et al., 2007). The variability between hyper and hypothyroid patients in pain thresholds, even when TSH levels are equivalent, indicates that it is either a T3/T4 effect or a secondary indirect effect.

Similarly, a correlation of headache to high or low TSH levels has not been consistent. In one group of patients, high TSH values were associated with low headache prevalence (Hagen et al., 2007). Other studies show TSH levels are normal in cluster headache patients, but there is a reduced TSH response to TRH during cluster periods (Waldenlind and Gustafsson, 1987; Bussone et al., 1988; Leone et al., 1990). It is unclear whether this decreased TSH surge is the result of amplified stress, altered hypothalamic aminergic-peptidergic regulation, endogenous depression or overproduction of TRH (Engler et al., 1982; Jackson, 1982; Loosen and Prange, 1982; Leone and Bussone, 1993). Multiple animal studies on TRH concluded it does not influence basal nociception, or have a complex action on morphine-induced anti-nociception (Watkins et al., 1986; Cridland and Henry, 1988).

TSHr is mainly expressed by small peptidergic sensory neurons (Table 1; Usoskin et al., 2015). THr-beta (T3 receptor) is expressed at low levels in DRG sensory neurons (Table 1), but THr-alpha (T3 and T4 receptor) is present in every DRG sensory neuronal group at substantial levels (Table 1; Usoskin et al., 2015). TRHr is almost absent in DRG neurons (Table 1; Usoskin et al., 2015). In clinical settings and behavioral experiments on animals, it is very difficult to determine whether effects of certain thyroid hormones are direct or indirect, since they not only control each other’s production and secretion, but they also regulate the production of other hormones as well as many immune cell functions. In total, published data on nociceptive effects of the thyroid system in animals are inconsistent and often contradict clinical observations on patients.

## Gonadotrophins

Gonadotrophins, LH and FSH regulate the production of steroid sex hormones (GnH) by the ovaries and testes. LH and FSH are heterodimers with alpha and beta subunits. The alpha subunits (92 AA) are identical in LH, FSH and TSH, leading to high levels of cross-reactivity between hormones and receptors. FSH and LH production and secretion are controlled by GnRH supplied by specialized hypothalamic neurons. GnRH is in turn strictly regulated (inhibited) by GnHs, especially estradiol and testosterone. LH and FSH are regulated by virtually every pituitary hormone via numerous pathways. Generally, these regulations occur either through GnRH or GnH pathways. Because of their diverse regulation, LH and FSH levels are often affected during pain conditions, especially chronic ones. However, clinical reports are not as consistent as basic research data. Some studies reported reduction of LH and increase in FSH basal levels in headache patients (Leone and Bussone, 1993). It was noted that this type of pattern is also observed in pre-pubescent and post-menopausal subjects who lack central control of sex hormone levels. Lack of such control is also observed in migraine and cluster headache patients (Leone and Bussone, 1993). In addition, discrepancies in clinical studies could be due to effects of anti-pain drugs and stress on LH and FSH levels (Rivier et al., 1986; Leone and Bussone, 1993). The male predominance in cluster headache patients along with the typical post-adolescence onset indicates a sex hormone role, particularly testosterone and LH dependent (Manzoni et al., 1991). Both LH and testosterone reductions in cluster headache patients could be pain-inducing events through either direct or indirect mechanisms (Micieli et al., 1987).

Modulation of nociceptive pathways by FSH and/or LH is mainly studied in association with steroid sex hormone regulation of this pathway. There is an agreement and enormous number of reports that GnH play a pivotal role in the perception of somatosensory stimuli, nociceptive transmission and pain chronicity (Traub and Ji, 2013). A recent report shows that male migraine patients exhibited elevated levels of estradiol and had clinical evidence of relative androgen deficiency (van Oosterhout et al., 2018). Reports on the roles of GnH in pain and nociception will not be covered here, since they are summarized in multiple excellent reviews, including: Gintzler and Liu (2012); Amandusson and Blomqvist (2013); Traub and Ji (2013). Nevertheless, despite the number of findings on regulation of nociceptive pathways by GnH, little is known on mechanisms governing these effects. They likely involve complex mechanisms, which encompass numerous neuroanatomical circuits, neuron-modulating pathways and messengers/factors controlling interaction of neurons with non-neuronal cells.

Regulation of the endogenous opioid system by GnH is one of the most studied areas of hormonal regulation of nociception (Gintzler and Liu, 2012); however, a majority of critical questions remain unanswered: what signaling pathway(s) controls neuronal plasticity within GnH-regulated nociception? How do GnHs interplay with other hormones, especially pituitary hormones closely regulated by GnH, and how do they regulate nociceptive transmission? Will local treatment with GnHs have the same effect on nociception/hypersensitivity as the massive changes observed after systemic replacement or deprivation of GnHs? Nevertheless, generation of transgenic lines and development of modern approaches such as optogenetics, RNAseq (both tissue and single cell) and detection of specific cell activities in *ex vivo* and *in vivo* preparations will undoubtedly help tackle many of these questions.

## Conclusion

It can conservatively be said that pituitary hormones control nearly all vital systems and physiological processes in mammals. For many decades, clinical characterization of pathological conditions has involved collecting hormone panels, including for many pain conditions. Data from these accumulated hormone panels show pain conditions correlate with altered serum levels of certain pituitary hormones, hypothalamic hormones controlling pituitary hormones and/or glandular hormones governed by pituitary hormones. Even with all the data, it is still not clear whether pain conditions lead to these hormonal changes or if hormonal imbalances trigger the pain conditions. This problem is especially precarious with regard to the many orofacial pain disorders that show age or sex-dependence because of the physiologic variance of pituitary hormones between sexes and developmental stages. The most pressing question explored in many of the referenced studies that still needs to be answered is, “Do pituitary hormones directly or indirectly modulate nociceptive pathways, pain threshold and chronicity?”

Research focused on pituitary hormones in pain transmission provides a number of challenges and advantages. First, as mentioned above, natural age and sex-dependency of hormones makes them challenging to correlate to pain disorders and also to translate between animal and humans. However, the sex differences mean hormones are viable mechanistic and therapeutic candidates for the sexual dimorphism seen in pain threshold and chronicity. The second challenge/advantage is that pituitary hormones are capable of controlling many cellular signaling pathways ranging from transient to chronic to epigenetic. The diverse mechanisms of action provide many targets to explore, but trying to narrow the pathways responsible for specific changes in nociception is difficult. The third challenge/advantage is that pituitary hormones are capable of not only endocrine, but also autocrine and paracrine actions. Again, the diversity of actions indicates hormones are viable targets for many habitus-wide pain states, but pinpointing mechanisms is daunting. The last challenge/advantage for studying pituitary hormones is the extremely complex cross-interaction between pituitary hormones as well as the downstream hormones they regulate. The vast overlap makes even developing the right questions overwhelming.

Even with their challenges, pituitary hormones remain targets for extensive research in many fields, including: neuroscience, immunology, reproductive biology, metabolism and cancer research. This is dictated by their critical involvement in many pathological conditions and their appeal as worthwhile “druggable targets.” As an example, currently-approved therapeutics include: GH, GHr antagonist (pegvisomant), Somatostatins, Dopamine agonists (to block PRL), TH (synthroid), FSH, LH, FSH/LH combos (menopur), FSH receptor antagonists, GnRH agonists and antagonists, ACTH, OXT and many more.

## Author Contributions

AA wrote the first draft of the manuscript. AA, JB and GD prepared final version of the manuscript.

## Conflict of Interest Statement

The authors declare that the research was conducted in the absence of any commercial or financial relationships that could be construed as a potential conflict of interest.
